# Reduction of density-modification bias by β correction

**DOI:** 10.1107/S0907444911002083

**Published:** 2011-03-18

**Authors:** Pavol Skubák, Navraj S. Pannu

**Affiliations:** a Biophysical Structural Chemistry, Leiden University, PO Box 9502, 2300 RA Leiden, The Netherlands

**Keywords:** reliable figure-of-merit estimates, density modification, maximum likelihood, bias reduction

## Abstract

Density modification often suffers from an overestimation of phase quality, as seen by escalated figures of merit. A new cross-validation-based method to address this estimation bias by applying a bias-correction parameter ‘β’ to maximum-likelihood phase-combination functions is proposed. In tests on over 100 single-wavelength anomalous diffraction data sets, the method is shown to produce much more reliable figures of merit and improved electron-density maps. Furthermore, significantly better results are obtained in automated model building iterated with phased refinement using the more accurate phase probability parameters from density modification.

## Abstract 2

A cross-validation-based method for bias reduction in ‘classical’ iterative density modification of experimental X-ray crystallography maps provides significantly more accurate phase-quality estimates and leads to improved automated model building.

## 1. Introduction

Density modification (DM) can significantly improve an electron-density map by incorporating features that are expected to appear in the map, such as flatness or disorder of the solvent region (Wang, 1985 ▶), the similarity of regions related by noncrystallographic symmetry (Bricogne, 1974 ▶) and the similarity of the density-map histogram to histograms of deposited macromolecules (Zhang & Main, 1990 ▶).

Errors are introduced when the experimental map is modified according to the expectations. The errors may have different sources, for example inaccurate identification of solvent regions from the experimental map or inaccurate noncrystallographic symmetry operators. In order to reduce the effect of the introduced errors, the modified map is recombined with the original experimental information and the resulting combined map is passed to the next cycle of density modification.

In order to combine the experimental and modified phases optimally, a likelihood function can be constructed for the estimation of errors in the experimental and modified phases and subsequent estimation of the combined phases. While the likelihood function of the experimental phases and a corresponding estimation of their errors is known from experimental phasing, the errors in the density-modified phases can be estimated from the agreement between the observed and modified amplitudes. Traditionally, the estimation is performed using the σ_A_ algorithm (Srinivasan & Ramachandran, 1965 ▶; Srinivasan, 1966 ▶; Read, 1986 ▶), where the σ_A_ parameter and the closely related Luzzati error parameter (Luzzati, 1952 ▶) are the estimated measures of accuracy of the model structure factors.

### 1.1. Bias in density modification

In order to obtain an unbiased estimation of a parameter from an agreement between the observations and the model, the model should be derived independently from the observations. However, the density-modified map is obtained from the experimental map, leading to an artificially high correlation between the observed and modified amplitudes. For example, in an extreme case of ‘null’ modification (Cowtan & Main, 1996 ▶), the density-modified map is equal to the experimental map and a perfect agreement exists between the null-modified and observed amplitudes. The σ_A_ and Luzzati error estimates then become much higher than their ‘true’ values and the errors in the null-modified phases would be estimated as much smaller than the errors in the experimentally derived phases although they are identical.

The underestimation of errors in the modified phases leads to suboptimal phase combination. The combined phases become biased towards the modified phases, which is referred to as model bias. Furthermore, it leads to statistical bias in the estimation of the resulting phase quality as the measure of combined phase quality, the figure of merit, becomes overestimated. Despite this distinction, the source of both types of bias is the same and a single term ‘bias’ will be used to describe the negative consequences of consistent underestimation of errors in the modified phases.

The probability distribution of combined phases is usually constructed by a multiplication of the experimental phases distribution by the distribution of model phases. However, the multiplication is equivalent to an assumption of independence of the two probability distributions. Clearly, this assumption is incorrect for the reasons explained above, which further amplifies the problem of bias in density-modification procedures.

### 1.2. Current bias-reduction methods

Several techniques have been developed to reduce the bias. The γ correction (Abrahams, 1997 ▶) can be applied to the modified map, aiming to subtract the contribution of the experimental structure factor from the modified structure factor, thus reducing the correlation between the experimental and model amplitudes. As a special case, γ correction leads to solvent flipping (Abrahams & Leslie, 1996 ▶) instead of solvent flattening.

Another widely used technique is the synthesis of a 2*mF*_o_ − *DF*_c_ map instead of a centroid *mF*_o_ map for the next cycle of density modification. It has been shown that the 2*mF*_o_ − *DF*_c_ map supresses electron-density peaks resulting from errors in the model, thus reducing the effect of model bias in the density map (Main, 1979 ▶; Read, 1986 ▶). Furthermore, the 2*mF*_o_ − *DF*_c_ map is less correlated with the experimental map than the centroid map, thus also reducing the correlation between the experimental and the modified structure factors in the next cycle.

‘Statistical density modification’ (Terwilliger, 1999 ▶, 2000 ▶; Cowtan, 2000 ▶) uses a different density-modification scheme from ‘classical density modification’ as described so far: based on the map expectations, a probability distribution of density is constructed instead of a single modified map. This distribution is then transformed to reciprocal space, where it is combined with the experimental probability distribution, assuming their independence, and the combined distribution is in turn used for a likelihood-based estimation of phases for the next cycle map. The assumption of independence may be better justified than in classical density modification as the probability distribution describing the map expectations is less influenced by the experimental data.

Recently, a phase-combination scheme which incorporates experimental phase information in the form of Hendrickson–Lattman (HL) coefficients (Hendrickson & Lattman, 1970 ▶) in the distribution of the modified phases (Cowtan, 2010 ▶; Pannu *et al.*, 1998 ▶) has been shown to outperform the σ_A_ phase combination traditionally used in classical density modification. Furthermore, incorporation of the experimental phase information employing multivariate statistics has also been implemented for single anomalous diffraction (SAD) experiments (Skubák *et al.*, 2010 ▶). Unlike the implementation using HL coefficients, the SAD function does not explicitly assume independence of the model and the observations. Although the independence assumption was considered to be a major cause of bias in classical density-modification algorithms (*e.g.* Cowtan, 1999 ▶; Abrahams, 1997 ▶), its removal by the SAD function only leads to a slight reduction in bias. This suggests that the correlation between the model and the observations, despite its decrease by current bias-reduction techniques, remains artificially large and is the major reason for bias in the current classical density-modification programs.

Several cross-validation approaches have been proposed previously to address the problem of correlation between the model and the observations. Roberts & Brünger (1995 ▶) suggested monitoring the bias by looking at the difference between *R* and *R*_free_ values. In another approach, the bias is removed by a complete cross-validation in which the reflections are divided into 10–20 groups and a single cycle of density modification is repeated with each group excluded in turn as a free set. The union of the free sets is then used in the synthesis of the next cycle map, which successfully reduces the bias (Cowtan & Main, 1996 ▶). However, the performance of the method is suboptimal as part of the data is always excluded from density modification and the method is slower since every cycle has to be repeated 10–20 times. Estimation of error parameters from a fixed free set (Cowtan & Main, 1996 ▶; Pannu & Read, 1996 ▶) removes the efficiency problem, but still permanently excludes part of the data from density modification and creates a new problem of obtaining reliable estimates of error parameters from just the cross-validation set. Below, we propose a cross-validation-based approach to estimate the artificial contribution to the correlation between the observed and model amplitudes and to apply an appropriate correction to the recently implemented likelihood functions for phase combination.

## 2. Methods

### 2.1. β-correction method

The recently introduced likelihood functions for phase combination (Cowtan, 2010 ▶; Skubák *et al.*, 2010 ▶) assume a Gaussian distribution of structure factors, with the covariance between the model and the observed structure factor defined as 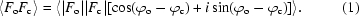
The imaginary part is small compared with the real part for a large number of reflections and can be omitted. As the observed phases ϕ_o_ are not known, the cosine term is usually estimated by a Luzzati error *D* parameter, which is either refined directly or estimated from a refined σ_A_ value, 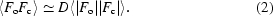
As discussed above, the 〈|*F*_o_||*F*_c_|〉 term is artificially large compared with other terms in the covariance matrix. Direct or indirect refinement of the *D* parameter against the working set of reflections cannot correct for the artificial increase and its refinement against the free set would mean permanent exclusion of part of the data from the density-modification procedure and potential reliability and stability problems. Therefore, we introduce a β error parameter which expresses the expected artificial increase in the correlation between |*F*_o_| and |*F*_c_| and is applied after refinement of the *D* parameter, 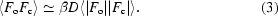
In our implementation, the β parameter is estimated using a simple cross-validation technique. The observations are divided into a free set and a working set and several cycles of density modification are performed using the working set of reflections. The β parameter is then estimated as the ratio of the covariance between the observed and the calculated structure-factor amplitudes of the free and working set of reflections, 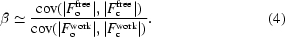
After β estimation, density modification is performed using all available observations, with the β parameter kept constant at its estimated value. In every cycle, the β parameter is applied after refinement of the Luzzati parameter by the likelihood function against all data. Although the β parameter can formally be considered as a correction to the Luzzati error parameter, their separation is essential in order to enable all observations to be used during refinement of the Luzzati parameter and during modification of the density.

### 2.2. Testing methodology

The method was implemented in the phase-combination program *MULTICOMB* (Skubák *et al.*, 2010 ▶) and tested on a wide range of real SAD data sets. The testing sample was the same as used in Skubák *et al.* (2010 ▶) and consisted of 102 data sets providing a wide range of resolution (from 0.94 to 3.29 Å) and anomalous scatterers, including selenium, sulfur, solvent molecules, bromides, calcium and zinc. The experimental maps for the density-modification programs were generated by the *CRANK* (Pannu *et al.*, 2011 ▶) structure-solution suite. *CRANK* performed substructure detection using either *AFRO* (Pannu *et al.*, unpublished work) and *CRUNCH*2 (de Graaff *et al.*, 2001 ▶) or *SHELXC* (Sheldrick, 2008 ▶), *SHELXD* (Schneider & Sheldrick, 2002 ▶) and *SHELXE* (Sheldrick, 2002 ▶). *BP*3 (Pannu & Read, 2004 ▶) was used for substructure phasing.

The performance and behaviour of the β-correction method was tested with two classical density-modification programs: *SOLOMON* (Abrahams & Leslie, 1996 ▶) from *CCP*4 (v.6.1.1; Collaborative Computational Project, Number 4, 1994 ▶) and *Parrot* (v.1.0.0; Cowtan, 2010 ▶) from *CCP*4 run within the *CRANK* suite. As *SOLOMON* and *Parrot* use different phase-combination, density-modification and bias-reduction algorithms, tests with both programs enable a better insight into the behaviour of the β-correction method.

For phase combination, *SOLOMON* employs the multivariate SAD–DM function as implemented in *MULTICOMB* and *Parrot* employs a Hendrickson–Lattman coefficient-based incorporation of experimental phase information. In order to test the β-correction method with *Parrot*, the internal *Parrot* phase combination was replaced by an external MLHL function (Pannu *et al.*, 1998 ▶) implemented in *MULTICOMB* which is based on the same theoretical principles and leads to negligible differences in *Parrot* performance (the difference in average map correlation was 0.004 and the correlation between the map correlations was 0.992 in tests on the specified sample of 102 data sets). Both programs make use of classical bias-reduction techniques: *SOLOMON* implements a theoretical γ correction, *Parrot* uses perturbation γ correction (Cowtan, 1999 ▶) and both programs use 2*mF*_o_ − *DF*_c_-type map synthesis.

The free reflections for the β estimation were selected randomly by *SFTOOLS* (B. Hazes, unpublished work) from *CCP*4, with the free set containing 5% of the total number of reflections for each data set. Five cycles of density modification were performed for β-parameter estimation, followed by 20 cycles of β-corrected density modification from the initial experimental map. Solvent flattening and histogram matching were used in all density-modification runs. Furthermore, automated noncrystallographic symmetry averaging as implemented in a development version (1.0.1) of *Parrot* was tested in §[Sec sec3.5]3.5.

The average statistical bias of the phase-quality estimation for the 102 data sets is calculated as 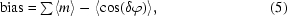
where the summation runs through all the data sets, 〈*m*〉 is the average figure of merit of a data set after density modification and δϕ is the difference between phase after density modification and phase calculated from a final deposited model for a reflection. The quality of a density-modified map is judged by its correlation with the map constructed from the deposited model, calculated by *SFTOOLS*. The map quality can also be judged by the automated model-building performance.

Either three cycles of *Buccaneer* (v.1.1.9; Cowtan, 2006 ▶) or ten cycles of *ARP*/*wARP* (v.7.1; Perrakis *et al.*, 1999 ▶) iterated with *REFMAC* (Murshudov *et al.*, 2011 ▶) were used for automated model building. The model-building performance is judged by the fraction of the model C^α^ atoms correctly built: a residue is regarded as ‘correct’ if its C^α^ atom is placed within 1 Å of a C^α^ position from the deposited model (*e.g.* Badger, 2003 ▶). The fraction of the model correctly built is calculated by a compare-protein script (Ness & Skubák, unpublished work) within the *CRANK* suite.

## 3. Results

### 3.1. Bias reduction

As shown in Table 1 ▶, the β-correction method strongly reduces the statistical bias of density-modified phase-quality estimation for both *SOLOMON* and *Parrot*. Furthermore, Table 1 ▶ indicates that both classical density-modification programs can produce less biased figures of merit than the statistical density-modification program *Pirate*.

The bias after *SOLOMON* is slightly smaller than the *Parrot* bias when the β correction is either used by both programs or not used by either of them. This is probably caused by the removal of the explicit assumption of independence by the SAD–DM function used by *SOLOMON*. However, the β correction is more important for bias reduction than removal of the assumption of independence.

**Figure 1:**
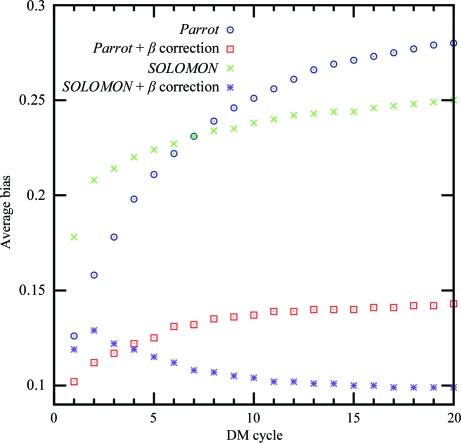
The average statistical bias of the sample of 102 data sets after each cycle of density modification with and without β correction by *Parrot* and *SOLOMON*.

The β correction reduces the statistical bias from the first cycle of density modification and the reduction increases in subsequent cycles, as shown in Fig. 1 ▶. With the β correction applied, the *Parrot* bias rises slowly in the first ten cycles and remains close to constant towards the end of density modification, while the average *SOLOMON* bias reaches its maximum in the second cycle and decreases afterwards. The reason for this behaviour is not known to us.

**Figure 2:**
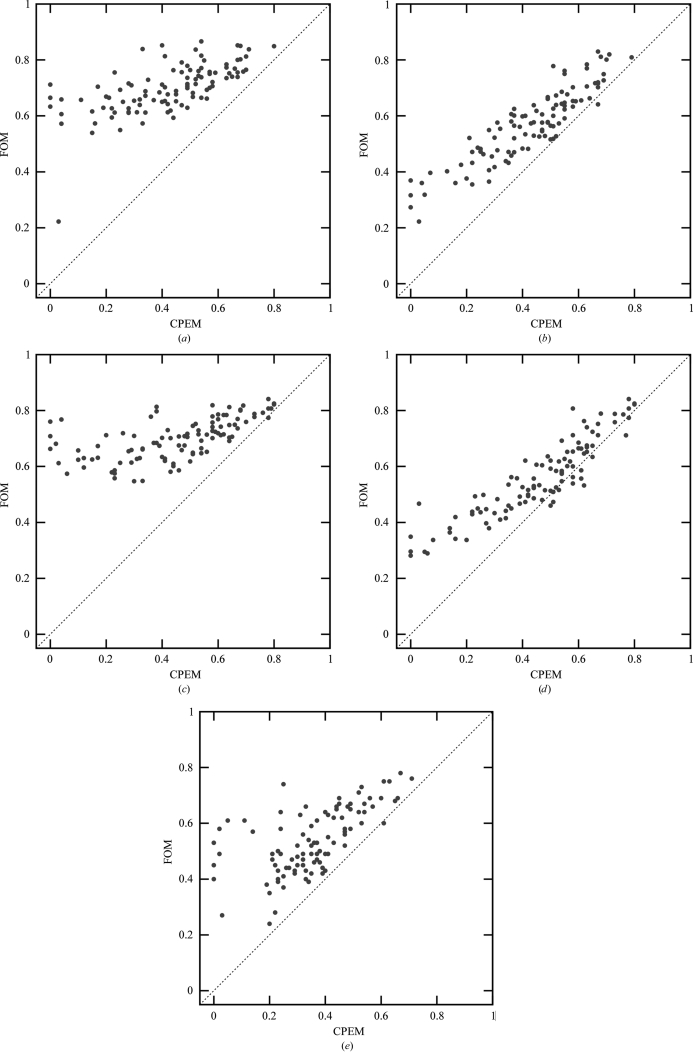
Average figure of merit of a data set as an estimator of the cosine of the mean phase error (CPEM) of a data set after (*a*) *Parrot* without β correction, (*b*) *Parrot* with β correction, (*c*) *SOLOMON* without β correction, (*d*) *SOLOMON* with β correction and (*e*) *Pirate*. The data point in the bottom left corner of (*a*) is an outlier caused by the *MULTICOMB* MLHL function minimizer becoming stuck.

**Figure 3:**
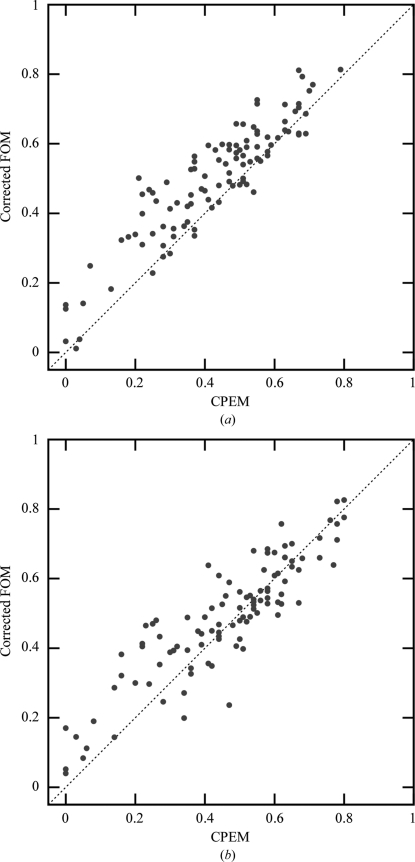
Average figure of merit corrected for bias after phasing *versus* cosine of the mean phase error (CPEM) for each data set after 20 cycles of β-­corrected density modification by (*a*) *Parrot* and (*b*) *SOLOMON*. The phasing bias-corrected figure of merit is defined as *m*_corr_ = *m* − [*m*_ph_ − cos(δϕ_ph_)], where *m* is the figure of merit after density modification, *m*_ph_ is the figure of merit after experimental phasing and δϕ_ph_ is the phase error after phasing.

Despite the improvements, the average statistical bias after density modification remains nonzero. In particular, data sets with low phase quality still suffer from an underestimation of the phase errors, as illustrated in Fig. 2 ▶. However, the phase quality of these data sets is typically overestimated by the previous phasing step. The almost symmetrical arrangement of data points around the diagonal in Fig. 3 ▶(*b*) shows that very little new bias is introduced during β-corrected density modification by *SOLOMON*. Thus, a method for bias reduction of experimental phase error estimation could lead to further improvements.

### 3.2. The figure of merit as phase-quality estimator

A precise estimation of the density-modified phase quality is essential for proper decision-making during or after density modification. Furthermore, density-modified phase probability statistics (*i.e.* Hendrickson–Lattman coefficients) can be used later in the structure-determination process. In the previous section, we have shown that β correction decreases bias in the estimation of phase quality by figure of merit. However, smaller bias of an estimator does not necessarily imply a better estimation of error owing to a potential bias–variance tradeoff.

The r.m.s. error of estimation of the mean cosine of the phase error of a data set by average figure of merit is summarized in Table 1 ▶. It shows that the β-correction method leads to significantly better phase-quality estimation for both *SOLOMON* and *Parrot* and surpasses the estimation by statistical density modification of *Pirate*. Furthermore, β correction does not introduce a bias–variance tradeoff as it also decreases the estimation variance. Fig. 2 ▶ provides a graphical representation of the improvements in bias, variance and error of the estimation.

The r.m.s. estimation error can be further decreased by performing a regression estimation of the relation between figure of merit and cosine of phase error. For each data set, we determined the shape of the regression curve by a nonparametric Nadaraya–Watson kernel regression (Nadaraya, 1965 ▶; Watson, 1964 ▶) using all data sets except the current data set. Such a leave-one-out cross-validated regression curve was used for estimation of the phase quality of the data set. A separate regression was performed for each of the density-modification programs with and without β correction. The r.m.s. error of the kernel regression estimation is determined by the variance of the distributions in Fig. 2 ▶. Although the kernel regression significantly decreases the estimation error, its practical use by density-modification programs is questionable since a reliable regression curve determined from tens or preferably hundreds of data sets would be needed for each density-modification program and for different sets of program options.

### 3.3. Map improvement from β-corrected density modification

Table 2 ▶ summarizes the effect of β correction on density-modification performance. On average, the quality of density-modified maps slightly improves if β correction is used, enabling better tracing of the structure by *Buccaneer*. The improvement can be attributed to model-bias reduction caused by correction of the underestimation of model phase errors. The performance gain is slightly better for *SOLOMON* compared with *Parrot*, which may be explained by stronger bias reduction in the case of *SOLOMON* using the SAD–DM function.

**Figure 4:**
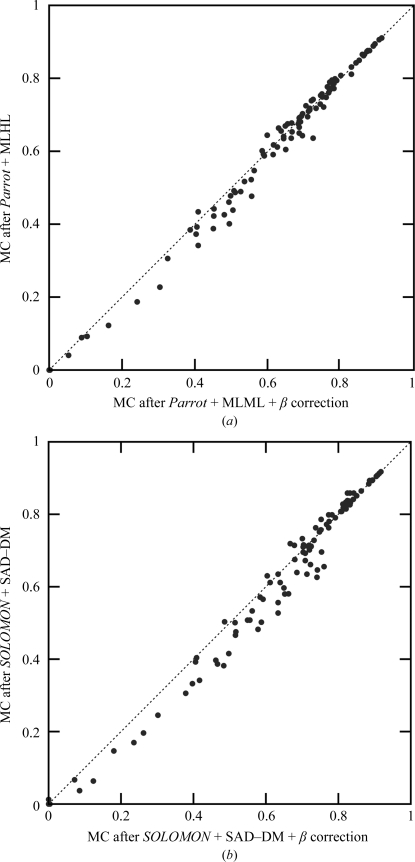
Average map correlation (MC) after density modification by (*a*) *Parrot* and (*b*) *SOLOMON* with β correction (*x* axis) and without β correction (*y* axis).

The performance depends on the quality of the density-modified map, as shown in Fig. 4 ▶. While maps with lower quality usually benefit from the correction, the quality of maps with a correlation with the deposited map higher than approximately 0.8 does not change significantly. This is owing to the little amount of bias in high-quality maps, as illustrated by a β parameter of close to one.

**Figure 5:**
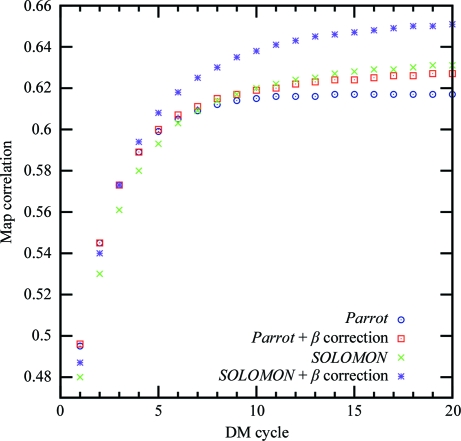
Improvement of map quality during density modification by *Parrot* and *SOLOMON* with and without β correction.

Classical density-modification programs often attempt to reduce the bias introduced by limiting the number of density-modification cycles. For example, the default number of cycles of *Parrot* is three. However, Fig. 5 ▶ shows that a preliminary end of the density-modification procedure can lead to significantly worse map quality. The use of β correction enables as many cycles to be used as needed for convergence of density modification, without a significant bias being introduced by multiple cycles (Figs. 1 ▶ and 6 ▶).

### 3.4. ’Null’ density modification

Although null density modification cannot improve the quality of the initial map, it is a useful validation method for bias-reduction techniques as it represents an extreme case of the greatest bias that can be introduced, with figures of merit typically rapidly approaching one after a few cycles of density modification. A good bias-reduction technique should be able to decrease the bias introduced during ‘null’ density modification and let the figures of merit converge closer to the real cosines of the phase error.

**Figure 6:**
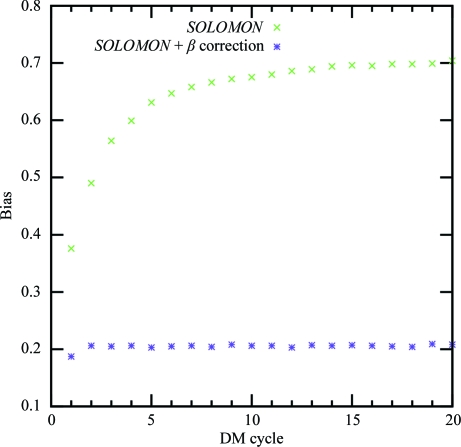
Average statistical bias after each cycle of ‘null’ density modification with and without β correction by *SOLOMON*.

Fig. 6 ▶ shows the development of the average statistical bias during *SOLOMON* density modification with and without β correction. Despite the γ correction, bias builds up rapidly with every cycle and reaches 0.7 after 20 cycles of density modification if the β correction is not used, which corresponds to figures of merit for all data sets of close to one. In contrast, the average bias in β-corrected ‘null’ density modification only rises slightly in the first two cycles and remains constant at approximately 0.2 during the rest of the procedure. ‘Null’ density modification by *Parrot* leads to similar results (data not shown).

### 3.5. β correction and NCS averaging

**Figure 7:**
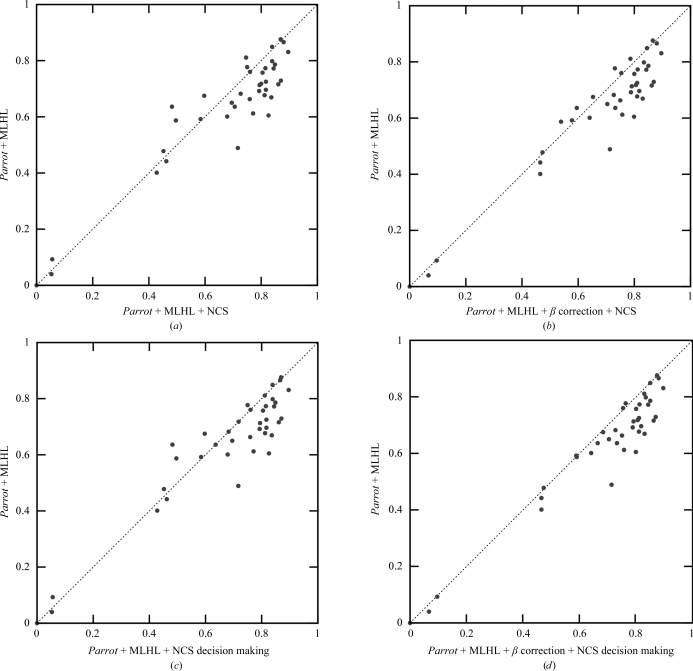
Correlation of a map constructed from a deposited model with the map after *Parrot* density modification without NCS averaging and without β correction (*y* axis) plotted against the map correlation after *Parrot* using NCS averaging (*x* axis) (*a*) without β correction, (*b*) with β correction, (*c*) with figure-of-merit-based decision-making and without β correction and (*d*) with figure-of-merit-based decision making and with β correction. Only the data sets for which *Parrot* determined NCS operators from the heavy-atom substructure are shown. Solvent flattening and histogram matching were used in all tests.

The previously discussed tests were performed without using information about noncrystallographic symmetry (NCS) in density modification. Fig. 7 ▶(*a*) shows the performance of *Parrot* with and without NCS averaging for 39 data sets for which NCS operators were automatically determined by *Parrot* from a heavy-atom substructure. On average, NCS averaging significantly improved the electron-density map quality. In a few cases the averaging led to worse maps (the points above the diagonal line), which turned out to be caused by incorrect determination of the NCS operators by *Parrot*. The errors introduced into the maps by averaging of regions not related by NCS are suppressed by β correction, while the quality of the maps for which correct NCS operators were identified remains approximately the same, as shown in Fig. 7 ▶(*b*).

Furthermore, we have tested whether figures of merit can be used to identify the data sets with incorrect NCS operators determined. Two separate density-modification runs with and without NCS averaging were performed for all data sets and the runs providing higher figures of merit were selected. Fig. 7 ▶(*d*) shows that all significant regressions caused by NCS averaging have been corrected by this decision-making. The use of β correction was essential for the successful identification of regression by figures of merit, as the decision-making was not reliable without it (Fig. 7 ▶ *c*).

**Figure 8:**
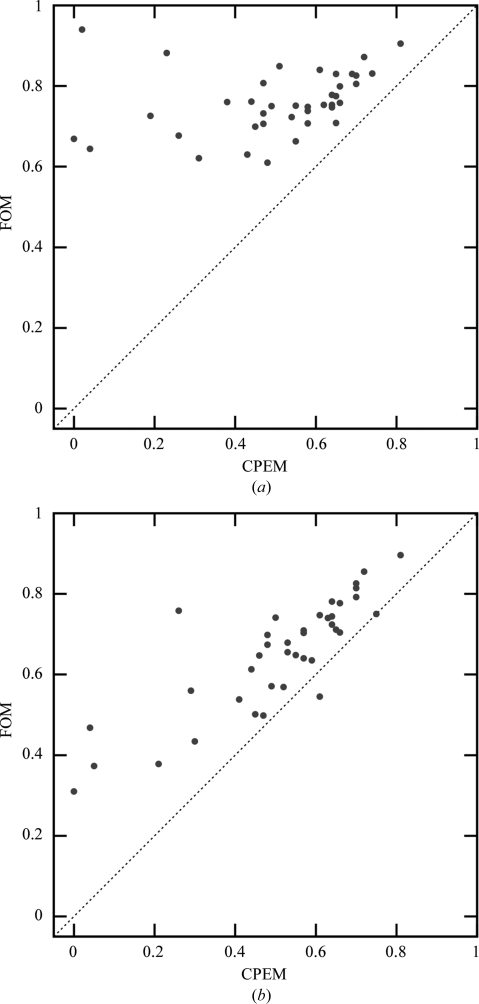
Average figure of merit of a data set *versus* the mean cosine of phase error (CPEM) of a data set after *Parrot* with NCS averaging (*a*) without β correction and (*b*) with β correction.

Fig. 8 ▶ shows that β correction leads to a significant decrease of the statistical bias of density modification with NCS averaging. The average statistical bias of the set of 39 data sets decreased from 0.251 to 0.142. However, the reduction of bias is slightly smaller compared with density modification of the same set of data sets without NCS averaging, where the average bias decreased from 0.266 to 0.125. This effect is probably caused by the relation between the free and the working set of reflections imposed by NCS averaging decreasing the reliability of β-parameter estimation. A possible workaround to this problem is the selection of free reflections from thin shells.

### 3.6. Subsequent use of phase probability distributions from density modification

The quality of phase probability distributions after density modification is especially important when these quantities are subsequently used in the structure-determination process, for instance in model building. We have tested the performance of model building by *ARP*/*wARP* iterated with *REFMAC* using different phase probabability distributions on all data sets. The results are summarized in Table 3 ▶.

The average fraction of the model correctly built increases if the previously determined Hendrickson–Lattman coefficients are incorporated in refinement by *REFMAC*’s MLHL target function compared with the Rice function, which does not use any information about experimental phases. However, on average there is hardly any improvement when using the Hendrickson–Lattman coefficients after density modification over the coefficients from experimental phasing because of the strong bias in the density-modified error estimates. The reduction of the bias owing to β correction enables automated building of data sets that fail otherwise, leading to a significant increase in the average fraction built. The trend is similar if the co­efficients are from either *Parrot* or *SOLOMON*.

## 4. Discussion

β correction has been shown to strongly reduce the statistical and model bias that occur in the classical density-modification programs *SOLOMON* and *Parrot*. The bias introduced in β-­corrected ‘classical density modification’ can be smaller than the bias introduced by ‘statistical density modification’, as shown by comparison with the program *Pirate*. The bias reduction is slightly better for *SOLOMON*, which can be attributed to the removal of the explicit assumption of independence by the SAD–DM phase-combination function used by *SOLOMON*. The majority of the statistical bias remaining after β-­corrected density modification by *SOLOMON* is not introduced in density modification but comes from experimental phasing.

The figures of merit after β-corrected density modification are significantly more accurate estimators of the quality of density-modified phases. This is important for decision-making during and after density-modification procedures. As an example, we have shown that β correction enables the identification of data sets with incorrect NCS operators used for NCS averaging. Futhermore, the improved quality of the density-modified phase probability distributions is important for subsequent use of phase probability parameters such as Hendrickson–Lattman coefficients in model building and refinement. Indeed, the use of β-corrected phase probability distributions by *REFMAC*’s MLHL target function significantly improves automated model building by *ARP*/*wARP* iterated with refinement by *REFMAC*.

Currently, classical density-modification programs often stop the density-modification process prematurely after a few cycles in an attempt to prevent bias developing in subsequent cycles. This premature end of density modification leads to suboptimal maps being obtained. β correction solves this problem as it enables the use of as many cycles as needed for convergence of density modification without the introduction of significant bias. Indeed, we have shown that the statistical bias can even decrease during the density-modification process in some cases and it remains approximately constant after the second cycle in the extreme case of ‘null’ density modification.

The bias reduction is slightly less effective if NCS averaging is performed. This can be attributed to less reliable cross-correlated β-parameter estimation caused by the relation between the free and working sets of reflections imposed by NCS averaging. Selection of free reflections from thin shells may help to improve the results further. However, random selection is still sufficient for significant reduction of the bias introduced during density modification using NCS averaging.

Density modification with β correction using a known β parameter is as fast as density modification without β correction. Thus, the only slowdown associated with the method is incurred by the few additional density-modification cycles required for the cross-validated estimation of the β parameter.

Although all of the tests in this paper were performed on SAD data sets, the method is not restricted to SAD data, as suggested by preliminary testing on MAD data sets. However, in general MAD data sets tend to provide better experimental phases and less density-modification bias, leading to the need for fewer and less powerful DM bias-reduction techniques.

The β-correction method attempts to model the artificial increase in correlation between the model and the data rather than removing it. Therefore, it does not replace the current methods for correlation reduction such as γ correction and 2*mF*_o_ − *DF*_c_-type map synthesis. Instead, it should be used in addition to these methods.

## Figures and Tables

**Table 1:** Average statistical bias as defined by (5), correlation between average figure of merit (FOM) of a data set and mean cosine of the phase error (CPEM) of a data set, r.m.s. error of direct estimation of CPEM by FOM and r.m.s. error of a cross-correlated kernel regression estimation of CPEM by FOM FOM and CPEM are calculated after 20 cycles of density modification by *Parrot* with *MULTICOMB* MLHL phase combination, by *SOLOMON* with *MULTICOMB* SAD–DM phase combination and by *Pirate* for all 102 data sets.

	*Parrot* + MLHL		*SOLOMON* + SAD–DM		
	Original	With β correction	Original	With β correction	*Pirate*
Average bias	0.280	0.143	0.250	0.099	0.181
Correlation of FOM and CPEM	0.650	0.901	0.618	0.904	0.621
R.m.s. estimation error	0.316	0.166	0.305	0.137	0.219
R.m.s. regression estimation error	0.145	0.079	0.173	0.083	0.126

**Table 2:** Average map correlation after density modification by *Parrot* and *SOLOMON* and average fraction of the model correctly built by *Buccaneer*

	*Parrot* + MLHL		*SOLOMON* + SAD–DM	
	Original	With β correction	Original	With β correction
Map correlation	0.617	0.627	0.631	0.651
Fraction built	0.609	0.624	0.612	0.666

**Table 3:** Average fraction of the model correctly built by *ARP*/*wARP* v.7.1 using different phase information in *REFMAC* reciprocal-space refinement The same map after density modification by *Parrot* or *SOLOMON* with β correction was used as input to *ARP*/*wARP* in all four tests.

	*Parrot* + MLHL	*SOLOMON* + SAD-DM
Rice: no phase information	0.549	0.587
MLHL with HL from phasing	0.598	0.629
MLHL with HL from DM	0.603	0.619
MLHL with HL from DM with β correction	0.651	0.680
